# Establishment of LIF-Dependent Human iPS Cells Closely Related to Basic FGF-Dependent Authentic iPS Cells

**DOI:** 10.1371/journal.pone.0039022

**Published:** 2012-06-13

**Authors:** Hiroyuki Hirai, Meri Firpo, Nobuaki Kikyo

**Affiliations:** 1 Stem Cell Institute, University of Minnesota, Minneapolis, Minnesota, United States of America; 2 Division of Hematology, Oncology and Transplantation, University of Minnesota, Minneapolis, Minnesota, United States of America; 3 Division of Endocrinology, Department of Medicine, University of Minnesota, Minneapolis, Minnesota, United States of America; Baylor College of Medicine, United States of America

## Abstract

Human induced pluripotent stem cells (iPSCs) can be divided into a leukemia inhibitory factor (LIF)-dependent naïve type and a basic fibroblast growth factor (bFGF)-dependent primed type. Although the former are more undifferentiated than the latter, they require signal transduction inhibitors and sustained expression of the transgenes used for iPSC production. We used a transcriptionally enhanced version of OCT4 to establish LIF-dependent human iPSCs without the use of inhibitors and sustained transgene expression. These cells belong to the primed type of pluripotent stem cell, similar to bFGF-dependent iPSCs. Thus, the particular cytokine required for iPSC production does not necessarily define stem cell phenotypes as previously thought. It is likely that the bFGF and LIF signaling pathways converge on unidentified OCT4 target genes. These findings suggest that our LIF-dependent human iPSCs could provide a novel model to investigate the role of cytokine signaling in cellular reprogramming**.**

## Introduction

Mouse pluripotent stem cells can be divided into naïve and primed pluripotent cells, depending on their level of pluripotency, patterns of gene expression, and the cytokines required to maintain an undifferentiated state (self-renewal) [1], [2], [3], [4], [5]. Naïve pluripotent stem cells, represented by embryonic stem cells (ESCs) and standard iPSCs, typically require a combination of LIF and BMP (bone morphogenetic protein) 2 or BMP4 for self-renewal, whereas primed pluripotent stem cells, such as epiblast stem cells (EpiSCs) [6], [7], require bFGF and transforming growth factor β/activin A signaling for self-renewal. Naïve pluripotent stem cells can form chimeric mice upon injection into a blastocyst. Primed pluripotent stem cells can form chimeric mice only at a very low frequency, if at all. Nonetheless, both cell types retain pluripotency as exhibited by teratoma formation after being injected into immunocompromized mice. Naïve pluripotent stem cells specifically express *Rex1* (Z*fp42*), *Stella (Dppa3*) and *Klf4* and have two active X chromosomes in female cells. Primed pluripotent stem cells specifically express *Fgf5* and *T* (Brachyury) and have only one active X chromosome in female cells. Morphologically, naïve pluripotent stem cells form dome-shaped colonies and can be cloned after single-cell dissociation; however, primed pluripotent stem cells demonstrate flat colonies and cannot proliferate well after single-cell dissociation due to apoptosis. It is currently thought that primed pluripotent stem cells represent a more differentiated state than naïve pluripotent stem cells, reflecting the developmental stage of the source cells from which each cell type is derived. ESCs are commonly established from the inner cell mass of blastocysts around 3.5 days post coitum (dpc). EpiSCs are typically prepared from epiblasts on 5.5–6.5 dpc [6], [7], although EpiSCs were recently established from blastocysts as well [8].

Unlike their mouse counterparts, human ESCs and iPSCs appear to be primed pluripotent stem cells [1], [2], [3], [4], [5]. These cells require bFGF for self-renewal, display flat colony morphology, and tend to enter apoptosis upon single-cell dissociation. In addition, one of two X chromosomes is generally inactive in female human ESCs and iPSCs, although this may be dependent on culture conditions [9], [10], [11], [12]. Instead of the commonly used bFGF, LIF has also been used to maintain self-renewal. LIF-dependent human ESCs and iPSCs have characteristics of naïve stem cells; however, they require for self-renewal chemical inhibitors against cell signaling pathways and sustained expression of the transgenes used to establish iPSCs unlike their mouse counterparts [13], [14], [15], [16]. For instance, LIF-dependent human iPSCs were established with inhibitors against extracellular signal-regulated kinase 1/2 (ERK1/2) and glycogen synthase kinase 3 (GSK3) in addition to constitutive expression of the transgenes *OCT4* plus *KLF4*, or *KLF2* plus *KLF4* [16]. Expression of these transgenes can be replaced with forskolin, which induces expression of *KLF2* and *KLF4*. It is unclear if human LIF-dependent iPSCs can be established and maintained in the absence of these inhibitors.

**Figure 1:**
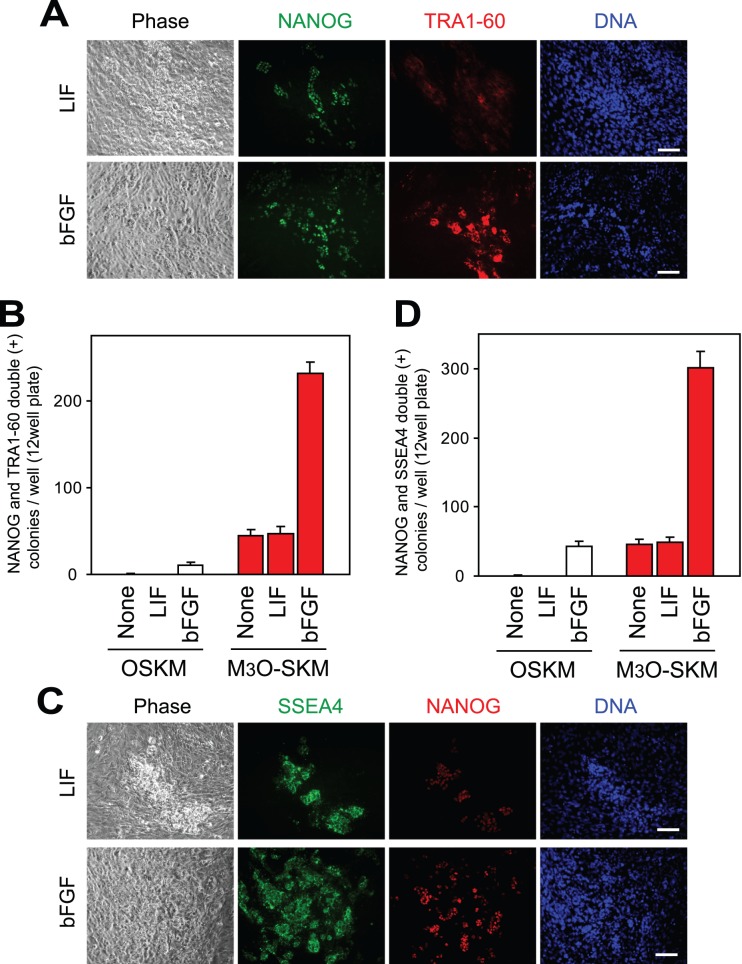
Expression of pluripotency markers by iPSCs prepared with LIF or bFGF. (A) Immunofluorescence staining of iPSCs prepared with LIF or bFGF on day 8. Antibodies against NANOG and TRA1-60 were used, and DNA was counterstained with Hoechst 33342. Bar, 100 µm in (A) and (C). (B) Formation of iPSC colonies that were double-positive for NANOG and TRA1-60 with LIF, bFGF, or no cytokines. Cells (1.7×10^4^) were seeded in each well, and iPSC colonies were counted on day 10. Mean + standard deviation (SD) was obtained from three independent experiments. (C) Immunofluorescence staining of iPSCs prepared with LIF or bFGF on day 8. Antibodies against SSEA4 and NANOG were used. (D) Formation of iPSC colonies that were double-positive for SSEA4 and NANOG with bFGF, LIF, or no cytokines. Cells were seeded and iPSC colonies counted as described in (B).

We recently established a novel strategy to significantly improve the efficiency of making mouse and human iPSCs by using a fusion protein, called M_3_O, between OCT4 and the powerful transactivation domain of the myogenic master transcription factor MYOD [17], [18]. M_3_O facilitates transcriptional activation by OCT4 without disrupting target gene specificity. M_3_O in combination with SOX2, KLF4 and c-MYC (M_3_O-SKM) increased the efficiency of iPSC colony formation by 10- to 50-fold and halved the time required for iPSC colony formation as compared with OCT4 and SKM (OSKM). Because of this high potency, we hypothesized that M_3_O-SKM might be capable of establishing human LIF-dependent iPSCs without having to utilize sustained transgene activation and chemical inhibitors.

**Figure 2:**
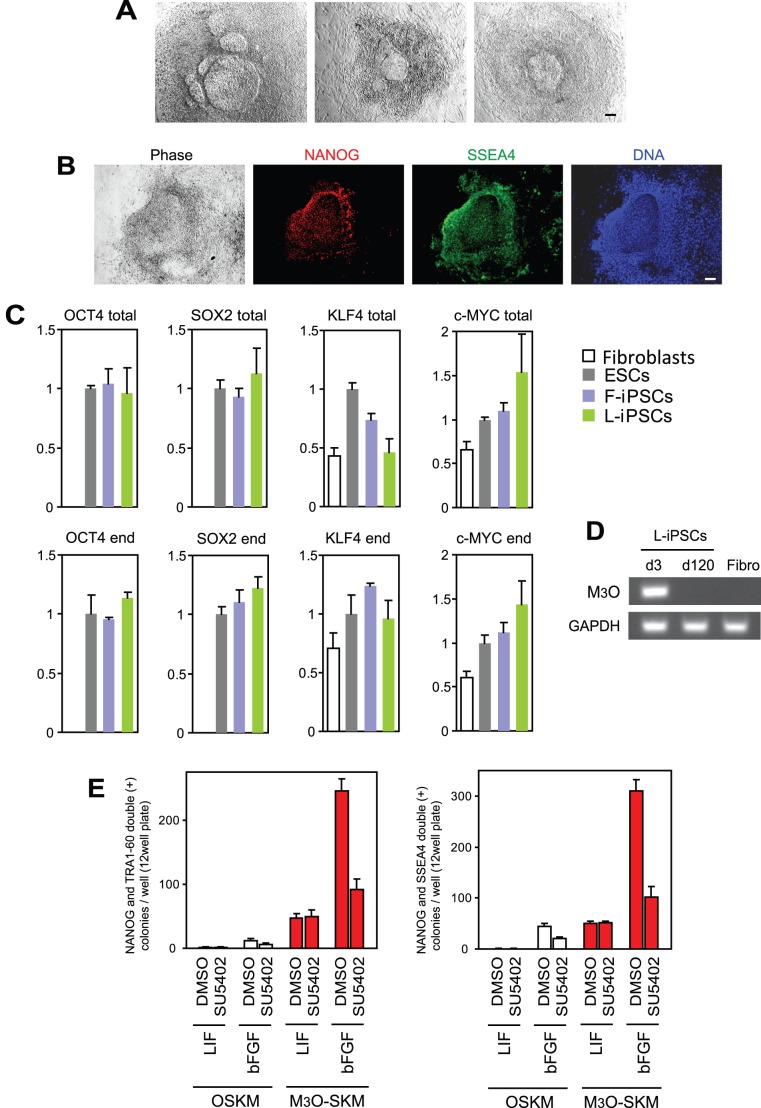
Characterization of colony morphology, gene expression patterns, and sensitivity to an FGF receptor inhibitor in L-iPSCs obtained with M_3_O-SKM. (A) Phase contrast images of L-iPSC colonies obtained on day 90. Bar, 100 µm. (B) Immunofluorescence staining of an L-iPSC colony with antibodies against NANOG and SSEA4 on day 28. Bar, 100 µm. (C) Quantitative RT-PCR analysis of the *OCT4*, *SOX2*, *KLF4* and c-*MYC* genes in various pluripotent stem cells on day 120. Expression levels of each gene were normalized to GAPDH. The normalized value for ESCs was defined as 1.0. Mean + SD obtained from three independent experiments are shown. (D) RT-PCR analysis of the expression of M_3_O in day-3 fibroblasts transduced with M_3_O-SKM, day-120 L-iPSCs, and parent fibroblasts. Expression of GAPDH mRNA was monitored as a control. Bar, 100 µm. (E) Effects of SU5402 on iPSC colony formation with LIF and bFGF. SU5402 dissolved in dimethyl sulfoxide (DMSO) was added to culture medium between day 8 and 10, and the colonies were counted on day 12. DMSO was used as a control.

## Results and Discussion

Adult human dermal fibroblasts were transduced with retroviruses expressing M_3_O-SKM or OSKM. Fibroblasts were subcultured onto feeder cells three days later, when addition of bFGF or LIF was also started. Control culture was prepared without bFGF and LIF. ESC-like colonies were defined as iPSC colonies when they were double-positive for the pluripotency markers NANOG and TRA1-60 (Fig. 1A). In the presence of bFGF, M_3_O-SKM induced iPSC colonies over 20 times more efficiently than OSKM on day 10 as previously reported [17], [18] (Fig. 1B). When bFGF was omitted or replaced with LIF, iPSC colonies were not observed with OSKM until day 16 at the earliest. However, iPSC colonies did appear around day 7 when M_3_O-SKM was used without either cytokine. Addition of LIF did not increase the efficiency of iPSC colony formation. When NANOG and SSEA4 were used as makers for iPSC colonies, similar results were obtained (Fig. 1C and 1D).

**Figure 3:**
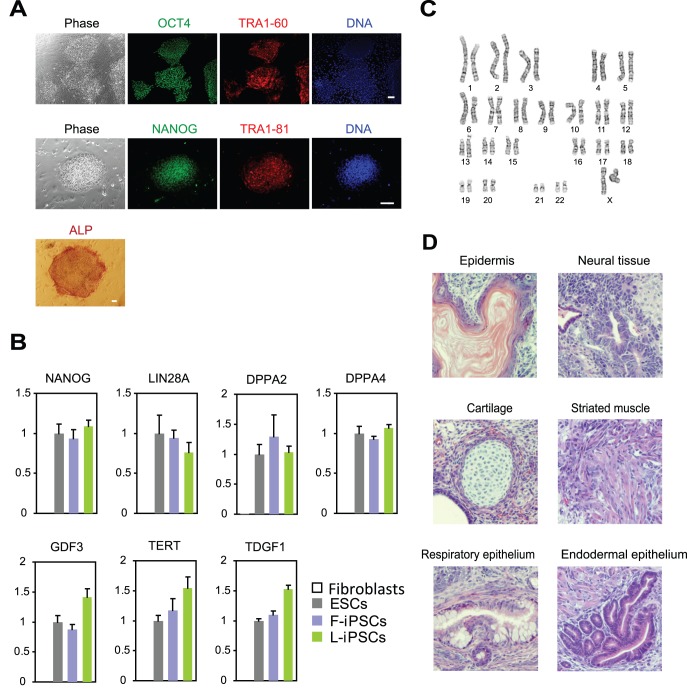
Characterization of gene expression patterns, karyotype, and teratoma formation in L-iPSCs prepared with M_3_O-SKM. (A) Immunofluorescence staining of L-iPSCs with antibodies against OCT4, NANOG, TRA1-60 and TRA1-81 on day 120. Alkaline phosphatase staining is also shown. Bar, 100 µm. (B) Quantitative RT-PCR analysis of genes that are typically expressed in pluripotent stem cells. Expression levels of each gene were normalized to GAPDH. The normalized value for ESCs was defined as 1.0. Mean + SD obtained from three independent experiments are shown. (C) Karyotype analysis of an L-iPSC on day 120. (D) Haematoxylin and eosin staining of histological sections of teratomas derived from L-iPSCs. Bar, 200 µm.

**Figure 4:**
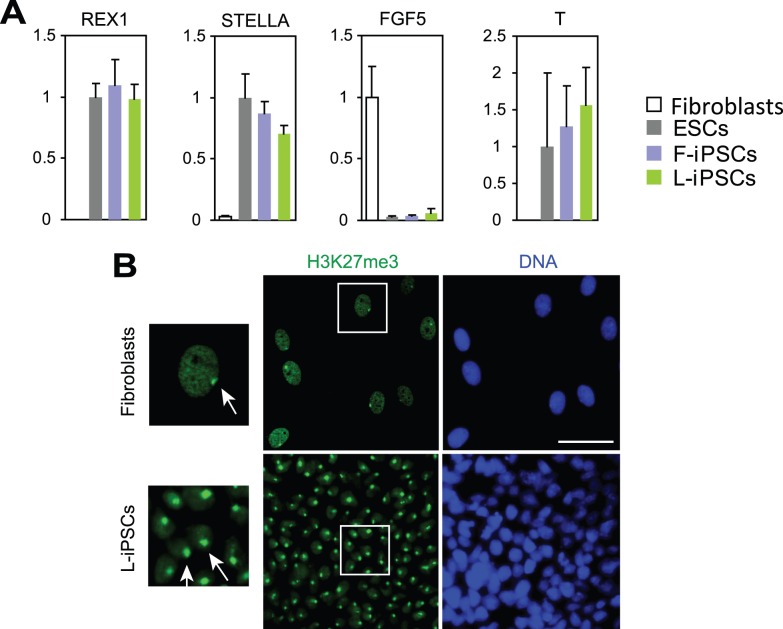
Characterization of L-iPSCs by using gene expression analyses and immunofluorescence staining of H3K27me3. (A) Quantitative RT-PCR analysis of genes that are typically expressed in mouse naïve (*REX1* and *STELLA*) or primed (*FGF5* and *T*) pluripotent stem cells. Expression levels of each gene were normalized to GAPDH. The normalized value for ESCs was defined as 1.0. Mean + SD obtained from three independent experiments are shown. (B) Immunofluorescence staining of H3K27me3 as an indicator for X chromosome inactivation. Parent fibroblasts were stained as a positive control. Nuclei surrounded by squares were magnified (left panels). Arrows indicate H3K27me-positive areas, corresponding to inactive X chromosomes. Bar, 50 µm.

We picked up 20–48 iPSC colonies from each culture condition between day 10 and 12 when colonies were available. These clones were then expanded on feeder cells in culture with the same cytokine to compare their efficiency of establishing pluripotent cell lines in the absence of signaling inhibitors. More than 90% of iPSC clones prepared with M_3_O-SKM in the presence of bFGF (F-iPSCs) remained undifferentiated for 120 days (25–30 passages) as monitored by the expression of TRA1-60 and NANOG. In the absence of bFGF and LIF, however, all iPSC colonies prepared with M_3_O-SKM morphologically differentiated by day 15 when the transgenes were suppressed although they remained undifferentiated if bFGF was added from day 15 onwards. In addition, most of the iPSC colonies prepared with M_3_O-SKM in the presence of LIF (L-iPSCs) differentiated around the same time. However, a few L-iPSC clones retained morphologically undifferentiated colonies scattered among numerous differentiated colonies beyond day 15. These apparently undifferentiated colonies were also unstable, with central undifferentiated parts being surrounded by differentiated derivatives (Fig. 2A). The undifferentiated state of the central parts was verified by the expression of NANOG and SSEA4 (Fig. 2B). The undifferentiated parts could be manually picked up during each subculture and expanded without substantial contamination with differentiated cells until day 200 at least.

**Figure 5:**
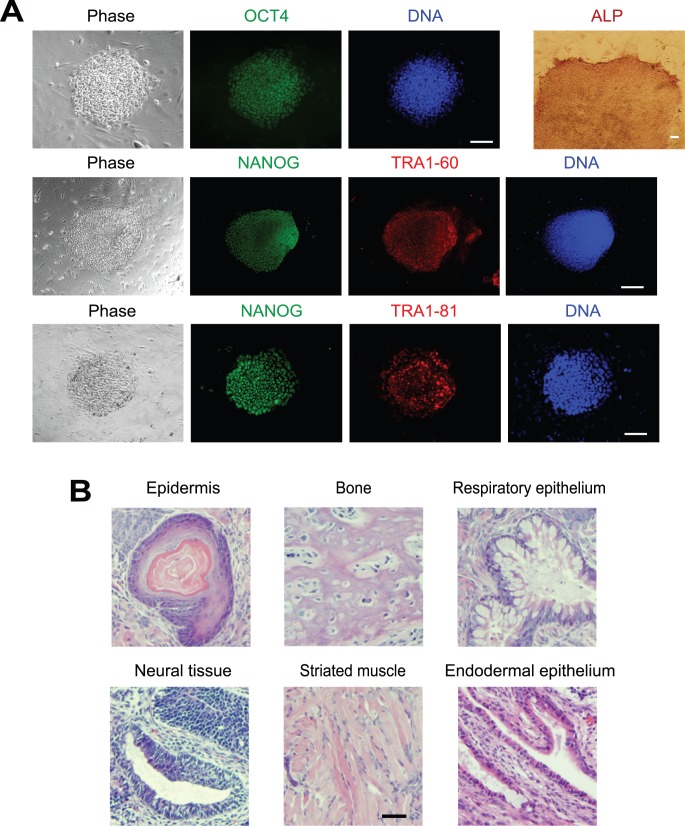
Characterization of LF-iPSCs with immunofluorescence staining and a teratoma formation assay. (A) Immunofluorescence staining and alkaline phosphatase staining of LF-iPSCs on day 140. Bar, 100 µm. (B) Haematoxylin and eosin staining of histological sections of teratomas derived from LF-iPSCs. Bar, 200 µm.

L-iPSCs established with this procedure were characterized with several approaches. First, suppression of the M_3_O-SKM transgenes was verified by comparing the levels of total mRNA (derived from endogenous genes and transgenes) encoding *OCT4*, *SOX2*, *KLF4* and c-*MYC* and those derived from endogenous genes. This was done using PCR primers specific to a 3′end untranslated region and coding region of each gene. Quantitative RT-PCR indicated that the relative expression levels of the four genes fell within a 2-fold range as compared with the levels in ESCs for both total and endogenous genes, suggesting suppression of the transgenes (Fig. 2C). Similar results were obtained with F-iPSCs. Suppression of the M_3_O transgene was also confirmed using a primer pair that spans the border between the M_3_ domain and *OCT4* (Fig. 2D).

**Figure 6:**
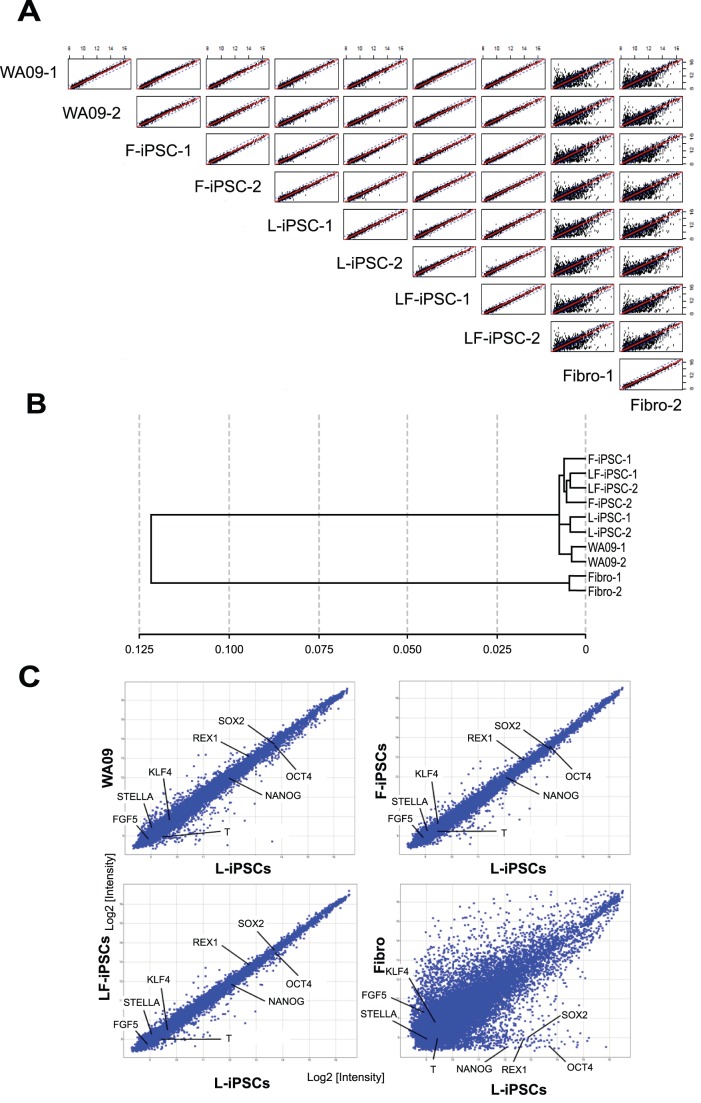
Genome-wide gene expression patterns comparing various pluripotent stem cells. (A) Scatter plots comparing levels of whole transcripts obtained from two WA09 ESC cultures, two dermal fibroblast cultures, two lines each for L-iPSCs, F-iPSCs and LF-iPSCs. Two independently cultured cells were used for WA09 cells. All these iPSCs were established from fibroblasts obtained from a single adult female. Two different cultures of fibroblasts obtained from the same individual were used. (B) Cluster analysis of the genome-wide gene expression patterns of cells analyzed in (A). (C) Scatter plots comparing levels of whole transcripts between L-iPSCs and other cell types used in this study. Average values obtained from two cultures or two lines for each cell type were used.

To rule out the possibility that autocrine bFGF was compensating for the lack of exogenous bFGF in L-iPSC culture, we blocked the FGF receptor with SU5402, an inhibitor of the FGF receptor tyrosine kinase [19] between day 8 and 10. The effectiveness of this treatment was verified by the observation that the number of F-iPSC colonies decreased to less than 40% of the control culture treated with the solvent dimethyl sulfoxide for both OSKM and M_3_O-SKM (Fig. 2E). However, SU5402 had no effect on the number of L-iPSC colonies, suggesting that bFGF did not play a predominant role in the formation of L-iPSC colonies. In addition, L-iPSCs cultured for 120 days expressed the pluripotency markers OCT4, NANOG, TRA1-60, TRA1-81 and alkaline phosphatase (Fig. 3A). Expression levels for seven additional genes enriched in pluripotent cells were similar to those in ESCs as shown by quantitative RT-PCR (Fig. 3B). Karyotype was normal in L-iPSCs (Fig. 3C). L-iPSCs also formed teratomas containing differentiated tissues derived from all three germ layers following injection into immunocompromized mice, proving pluripotency of these cells (Fig. 3D). L-iPSCs required the continuous presence of LIF for self-renewal and could not form ESC-like colonies after subculture without LIF.

To understand whether L-iPSCs belonged to the naïve or primed type, we compared them with F-iPSCs, which are primed pluripotent stem cells. First, L-iPSC colonies were flat and morphologically indistinguishable from F-iPSC colonies. Second, L-iPSCs did not form colonies after single-cell dissociation with trypsin during subculture, similar to F-iPSCs. Third, F-iPSCs and L-iPSCs expressed *REX1* (Z*FP42*), *STELLA (DPPA3*), *FGF5* and *T* at similar levels (Fig. 4A). Although *FGF5* is known to be expressed in primed pluripotent stem cells as described above, its expression level was much lower than that in fibroblasts. Fourth, the status of X chromosome inactivation was investigated by staining L-iPSCs with an antibody against trimethylation of lysine 27 on histone H3 (H3K27me3), whose enrichment is commonly observed in inactive X chromosome. Generally, only one X chromosome is active in female human F-iPSCs [9]. Almost all L-iPSCs showed one H3K27me3-positive area in each nucleus similar to the original fibroblasts, suggesting that one of the X chromosomes was inactivated in L-iPSCs (Fig. 4B). On the basis of the high similarity between F-iPSCs and L-iPSCs, we concluded that L-iPSCs belonged to the primed type pluripotent stem cell.

The similarity between F-iPSCs and L-iPSCs was further tested by examining if LIF in the culture medium of L-iPSCs could be replaced with bFGF on day 90 without disrupting self-renewal. After culture for an additional 50 days, the colonies, now called LF-iPSCs, maintained an ESC-like morphology and expressed OCT4, NANOG, TRA1-60, TRA1-81 and alkaline phosphatase (Fig. 5A). This cytokine switch diminished the tendency for differentiation; LF-iPSC colonies could be maintained in an undifferentiated state more easily than L-iPSCs. LF-iPSCs also preserved pluripotency as shown by teratoma formation following injection into immunocompromized mice (Fig. 5B). In an opposite experiment, bFGF was replaced with LIF for F-iPSCs, but the cells did not form any ESC-like colonies after subculture. Thus, unlike F-iPSCs, the pluripotency of L-iPSCs could be sustained by both the LIF and bFGF signaling pathways.

Finally, genome-wide transcription patterns were compared among ESCs, F-iPSCs, L-iPSCs, LF-iPSCs and parent fibroblasts with a microarray analysis. Overall gene expression patterns were quite similar among ESCs, two F-iPSC clones, two L-iPSC clones and two LF-iPSC clones (Fig. 6A). Hierarchical cluster analysis of genome-wide expression profiles demonstrated that L-iPSCs, F-iPSCs, LF-iPSCs and ESCs were very closely clustered (Fig. 6B). mRNAs encoding *OCT4*, *SOX2*, *NANOG*, *KLF4*, *REX1*, *STELLA*, *FGF5* and *T* were all expressed at similar levels among these pluripotent stem cells, with any differences being less than 1.5-fold (Fig. 6C).

Our study established L-iPSCs in the absence of sustained overexpression of transgenes and signaling inhibitors, unlike previously reported methods of making LIF-dependent human iPSCs. This was possible because of the usage of the transcriptionally augmented M_3_O. Reflecting differences in methods, L-iPSCs were also phenotypically different from previously reported LIF-dependent iPSCs and more closely related to standard bFGF-dependent iPSCs. This result has three important implications for our understanding of the regulation of pluripotency and self-renewal in iPSCs. First, dependency on LIF or bFGF is not the primary determinant of naïve or primed pluripotency. The activity of transgenes and modulations to cytokine signaling systems appear to be more critical in determining the type of pluripotent stem cell. Second, human and mouse cells are fundamentally different in terms of their response to LIF because this cytokine induces different types of pluripotent stem cells: primed-type human iPSCs and naïve-type mouse iPSCs [18]. Third, this study suggests that the diverse signaling pathways of LIF and bFGF are potentially converged at the level of OCT4-target genes. LIF regulates its target genes primarily through activation of the JAK kinase and resulting phosphorylation and DNA binding of STAT3 [3]. The main signaling pathway for bFGF is activation of MEK/ERK, leading to DNA binding of a variety of transcription factors [20]. There are no well-established overlapping target genes between these two signaling systems. In future studies, a detailed comparison of the target genes of OCT4 and M_3_O could potentially shed light on this novel mechanism underlying pluripotency and self-renewal.

## Materials and Methods

### Ethics Statement

All animal experiments were approved by the Institutional Animal Care and Use Committee at the University of Minnesota (1002A78174).

### Preparation of Human iPSCs

Human M_3_O, *OCT4*, *SOX2*, *KLF4* and c-*MYC* genes inserted in the pMXs-IP vector (Addgene) were individually transfected into Plat-A cells (Cell Biolabs) with Lipofectamin 2000 (Life Technologies) on day -3. On day -2, 2.7×10^4^ abdominal skin fibroblasts isolated from a 34-year-old female (Cell Applications) were plated in a well of a 12-well plate in DMEM with 10% FBS. On day -1 and day 0, virus supernatant was harvested from Plat-A cells and transduced each day into fibroblasts with polybrene after filtration through a 0.45 µm syringe filter. On day 3, transduced cells were subcultured at 1.7×10^4^ cells/well onto irradiated mouse embryonic fibroblasts on 12-well plates. The culture medium contained KnockOut DMEM/F-12, 20% KnockOut Serum Replacement, 1% insulin-transferrin-selenium, 1x GlutaMAX (all from Life Technologies), 100 µM MEM non-essential amino acids, 0.1 mM 2-mercaptoethanol, and either 4 ng/ml bFGF or 1,000 u/ml LIF. Culture medium was changed every two days. SU5402 (EMD Biosciences) dissolved in dimethyl sulfoxide (DMSO) was added at 5 µM into the culture medium between day 8 and 10. The human ESC line WA09 (WiCell) was cultured with 4 ng/ml bFGF in the culture medium used for iPSCs described above.

### Immunofluorescence Staining and Alkaline Phosphatase Staining

Following fixation with 4% formaldehyde in phosphate-buffered saline for 10 min, iPSCs were treated with 0.5% Triton X-100 for 3 min to permeabilize the plasma membrane. Primary and secondary antibodies (Table S1) were applied for 1 hour each at 25°C, followed by DNA counterstain with Hoechst 33342. An AxioCam charge coupled device camera was used to capture fluorescence images on an Axiovert 200 M fluorescence microscope (all from Carl Zeiss) equipped with a 10x A-Plan Ph1 Var1 objective (numerical aperture 0.25). Photoshop 7.0 (Adobe Systems) was used to process images. A Millipore kit (SCR004) was used for alkaline phosphatase staining following the manufacturer’s instructions.

### Quantitative RT-PCR (qRT-PCR)

Total RNA was prepared from each iPSC colony with a PureLink RNA Micro Kit (Life Technologies). cDNA was synthesized using a SuperScript III First-Strand Synthesis System for RT-CR (Life Technologies) and applied for qRT-PCR with a GoTaq qPCR Master mix (Promega) on a Realplex 2S system (Eppendorf). Primers are listed in Table S2. The mRNA encoding glyceraldehyde 3-phosphate dehydrogenase (GAPDH) was used for normalization.

### DNA Microarray Analysis

RNA was prepared from WA09 ESCs, the dermal fibroblasts from which all iPSCs in the microarray study were derived, two F-iPSC clones, two L-iPSC clones and two LF-iPSC clones on day 100–140 with the PureLink RNA total RNA purification system (Life Technologies). RNA quality was evaluated with a NanoDrop 8000 and an Agilent Bioanalyzer 2100. Biotin-labeled cRNA was synthesized with a TotalPrep-96 RNA Amplification Kit (Life Technologies/Applied Biosystems) following the manufacturer’s instructions. cRNA was hybridized onto a HumanHT-12 v.4.0 Expression Beadchip (Illumina) following the Direct Hybridization Gene Expression protocol. The Beadchips were then scanned using an Illumina’s iScan instrument. Data were created using Illumina’s GenomeStudio software and processed with Spotfire DecisionSite for Functional Genomics software. DNA microarray data have been deposited in the NCBI GEO database under the accession number GSE37077.

### Karyotyping

Colcemid-arrested iPSCs were treated with 0.75 M KCl hypotonic solution, and fixed with methanol and acetic acid at a 3∶1 ratio. Cell spreads were stained with Wright-Giemsa on glass slides for G band analyses with the Applied Spectral Imaging software.

### Teratoma Formation

NOD/SCID mice were injected with 1–2×10^6^ iPSCs into the hind limb muscle. Seven to eight weeks later, teratomas were isolated, fixed with 10% formalin and embedded with paraffin. Histological sections (5 µm thick) were prepared and stained with haematoxylin and eosin.

## Supporting Information

Table S1 **Antibodies used for immunofluorescence staining.**
(DOC)

Table S2 **Primers used for RT-PCR.**
(DOC)
