# Supplementary material for: Establishment of LIF-Dependent Human iPS Cells Closely Related to Basic FGF-Dependent Authentic iPS Cells
Source: PLoS One. 2012 Jun 13;7(6):e39022. doi: 10.1371/journal.pone.0039022 (PMC3374774; doi:10.1371/journal.pone.0039022)
Supplement: Table S1 — Antibodies used for immunofluorescence staining. (DOC) [file pone.0039022.s001.doc]

**Table S1. Antibodies used in immunofluorescence staining**

**Primary antibodies**

| **Antigen** | **Manufacturer** | **Catalog #** |
| --- | --- | --- |
| OCT4  NANOG  NANOG  SOX2  SSEA4, FITC-labeled  TRA-1-60, PE-labeled  TRA1-81, PE-labeled | Santa Cruz Biotechnology  R&D Systems  Abcam  Santa Cruz Biotechnology  BD Biosciences  BD Biosciences  BD Biosciences | sc-8628  AF1997  ab21624  sc-17320  560126  560193  560161 |

**Secondary antibodies**

| **Name** | **Manufacturer** | **Catalog #** |
| --- | --- | --- |
| PE-labeled anti-mouse Ig(M+G)  Alexa Fluor 488-labeled anti-mouse IgG Alexa Fluor 488-labeled anti-rabbit IgG  Alexa Fluor 488-labeled anti-goat IgG  Alexa Fluor 555-labeled anti-mouse IgG  Alexa Fluor 555-labeled anti-rabbit IgG  DyLight488-labeled anti-goat IgG  Cy3-labeled anti-goat IgG | BD Biosciences  Invitrogen  Invitrogen  Invitrogen  Invitrogen  Invitrogen  Jackson ImmunoResearch  Jackson ImmunoResearch | 550589  A11029  A11304  A11055  A21424  A21429  805-485-180  805-165-180 |
