# Supplementary material for: Establishment of LIF-Dependent Human iPS Cells Closely Related to Basic FGF-Dependent Authentic iPS Cells
Source: PLoS One. 2012 Jun 13;7(6):e39022. doi: 10.1371/journal.pone.0039022 (PMC3374774; doi:10.1371/journal.pone.0039022)
Supplement: Table S2 — Primers used for RT-PCR. (DOC) [file pone.0039022.s002.doc]

**Table S2. Primers used for RT-PCR**

| **Gene** | **Forward** | **Reverse** |
| --- | --- | --- |
| OCT4 total | AGCGAACCAGTATCGAGAAC | TTACAGAACCACACTCGGAC |
| SOX2 total | AGCTACAGCATGATGCAGGA | GGTCATGGAGTTGTACTGCA |
| KLF4 total | TCTCAAGGCACACCTGCGAA | TAGTGCCTGGTCAGTTCATC |
| c-MYC total | ACTCTGAGGAGGAACAAGAA | TGGAGACGTGGCACCTCTT |
| OCT4 endogenous | **CCTCACTTCACTGCACTGTA** | **CAGGTTTTCTTTCCCTAGCT** |
| SOX2 endogenous | **CCCAGCAGACTTCACATGT** | **CCTCCCATTTCCCTCGTTTT** |
| KLF4 endogenous | **GATGAACTGACCAGGCACTA** | **GTGGGTCATATCCACTGTCT** |
| c-MYC endog. | **TGCCTCAAATTGGACTTTGG** | **GATTGAAATTCTGTGTAACTGC** |
| NANOG | **TGAACCTCAGCTACAAACAG** | **TGGTGGTAGGAAGAGTAAAG** |
| LIN28 | GAGCATGCAGAAGCGCAGATCAAA | TATGGCTGATGCTCTGGCAGAAGT |
| DPPA2 | AGGCTTCATAGGCATGCTTACCCT | TGAAGCCTTGCTCTCTTGGTCACT |
| DPPA4 | AGACACAGATGGTTGGGTTCACCT | TGCACTCACTCTCCCTTCTTGCTT |
| GDF3 | ACACCTGTGCCAGACTAAGATGCT | TGACGGTGGCAGAGGTTCTTACAA |
| TERT | TGTGCACCAACATCTACAAG | GCGTTCTTGGCTTTCAGGAT |
| TDGF1 | TGCCCAAGAAGTGTTCCCTGTGTA | AAAGTGGTAGTACGTGCAGACGGT |
| REX1 | TGAATAGCTGACCACCAGCACACT | ACAGGCTCCAGCCTCAGTACATTT |
| STELLA (DPPA3) | CGAATCTGTTTCCCCTCTATCG | CTCTCCTGCTGTAAAGCCACTC |
| FGF5 | TGCAAGTGCCAAGTTCACAGA | **AGTTCTATGTATTGCTGAGGCATAGGTA** |
| T | TGCTTCCCTGAGACCCAGTT | GATCACTTCTTTCCTTTGCATCAAG |
| M_3_O | CGCTTCTTCGAAGACCTGGACC | CGAGAAGGCGAAATCCGAAGCC |
| GAPDH | **AACAGCGACACCCACTCCTC** | **CATACCAGGAAATGAGCTTGACAA** |
